# Evaluation of the telephone intervention in the promotion of diabetes self-care: a randomized clinical trial**^1^**

**DOI:** 10.1590/1518-8345.0632.2719

**Published:** 2016-08-29

**Authors:** Bárbara Sgarbi Morgan Fernandes, Ilka Afonso Reis, Heloisa de Carvalho Torres

**Affiliations:** 2 Doctoral Student, Escola de Enfermagem, Universidade Federal de Minas Gerais, Belo Horizonte, MG, Brazil.; 3 PhD, Adjunct Professor, Instituto de Ciências Exatas, Universidade Federal de Minas Gerais, Belo Horizonte, MG, Brazil.; 4 PhD, Associate Professor, Escola de Enfermagem, Universidade Federal de Minas Gerais, Belo Horizonte, MG, Brazil.

**Keywords:** Education, Nursing, Preceptorship, Education, Higher, Learning, Clinical Clerkship, Concept Formation

## Abstract

**Objective:**

to evaluate the effectiveness of the telephone intervention for promoting self-care related to physical activity and following a diet plan in users with diabetes, compared to conventional monitoring of users over a six-month period.

**Method:**

this was a randomized clinical trial, which included 210 users with diabetes, linked to eight Primary Health Units of Belo Horizonte, Minas Gerais. The experimental group (104 members) received six telephone interventions over the six-month monitoring; the control group (106 members) received conventional monitoring. To evaluate the self-care practices related to physical activity and following a healthy eating plan, in both groups, the self-care questionnaire was applied before the intervention and at three and six months after its start.

**Results:**

the mean effect of self-care scores in the experimental group was 1.03 to 1.78 higher than the control group, with progressive and significant improvement (p<0.001).

**Conclusion:**

the results indicate that the telephone intervention had a beneficial effect on diabetes self-care. The primary identifier of the clinical trials registry was: RBR-8wx7qb.

## Introduction

The complexity of the treatment, the complications and the increasing incidence of type 2 diabetes mellitus, associated with the behavior adopted by the user with diabetes, have caused health workers to mobilize in search of educational alternatives for the promotion of self-care directly related to physical activity and following a diet plan^1^ ^-^ ^3^. Accordingly, the telephone intervention has been used as an innovative educational strategy, being considered an effective form of communication by the professional and user^4^ ^-^ ^6^. This is because, through the use of understandable language, adapted to the reality in which the users and their needs in relation to self-care are the primary focus, the professional is able to negotiate, motivate and encourage them to take responsibility for their self-care^6^.

A recent integrative literature review, conducted with 40 experimental and quasi-experimental studies performed between 2008 and 2013, showed that the telephone intervention has a positive impact on improving glycohemoglobin levels and self-care in diabetes^7^. Complementarily, a randomized clinical trial conducted with 81 users with type 2 diabetes mellitus, carried out in Iran in 2012, showed that the group that received the telephone intervention for 3 months presented significant improvement in issues such as knowledge, attitudes, self-care and glycemic control, when compared with the control group^8^. Given this scenario, the telephone intervention was carried out in the Primary Health Units (PHUs) of Belo Horizonte, Brazil, aiming to promote education for diabetes self-care, valorizing aspects such as the autonomy of users regarding their choices, decision-making and the development of a care plan aimed at the fulfillment of goals^9^.

It was assumed that the evaluation of the telephone intervention would provide relevant subsidies for effective educational practice, allowing future implementations in diabetes education and representing a new field of practice for health professionals. Therefore, this study aimed to evaluate the effectiveness of the telephone intervention for promoting self-care related to physical activity and following a diet plan, in users with diabetes, compared to conventional monitoring of users over a six-month period.

## Methods

### Type and location of the study

This was a randomized clinical trial, conducted in eight Primary Health Units of the Eastern District of Belo Horizonte, Minas Gerais, Brazil.

### Participants

The study included users who were diagnosed with diabetes, aged 30 to 80 years, due to the higher prevalence of the disease in this age group^10^, had access to fixed or mobile telephones and participated in at least two telephone interventions. Users diagnosed with Type I diabetes, who did not answer the calls after five consecutive attempts and that changed address and phone number during the study were excluded.

### Intervention

The telephone intervention was carried out in a systematic way, aiming to promote self-care related to the practice of physical activity and following a diet plan, from the development of a plan to achieve the targets.

**Figure 1:**
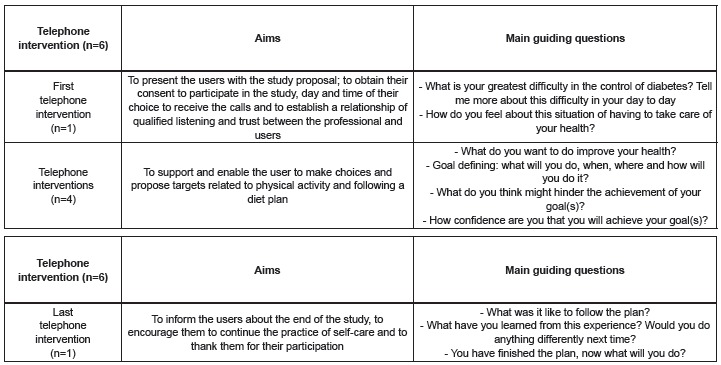
Telephone interventions performed according to their goals. Belo Horizonte, MG, Brazil, 2012

**Figure 2:**
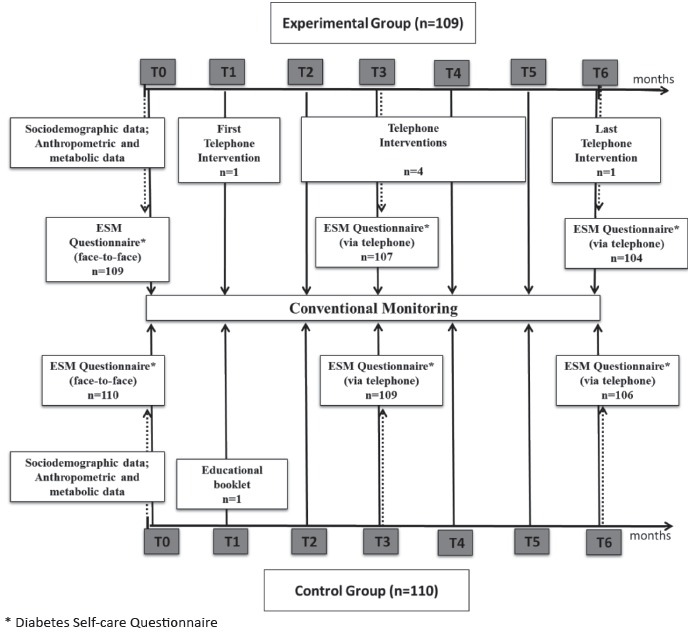
Graphical representation of the monitoring and/or intervention performed for the control and experimental groups. Belo Horizonte, MG, Brazil, 2012

As shown in Figure 2, the telephone interventions were conducted monthly by a nurse during the period from February to August 2012, in the Nursing School of the Federal University of Minas Gerais, with a mean of six telephone calls per user. The telephone interventions followed a previously established protocol and were classified into three types, according to their purpose, as described in Figure 1.

The care guidance followed the guidelines of the Brazilian Society of Diabetes (SBD) and were adapted according to the context and the actual conditions of the user^11^. In each telephone intervention the professional sought to enable the users to practice self-care and achieve the targets and to demonstrate to them that their participation and responsibility in the daily care could improve their health, encouraging them to establish one to two targets during each phone call^9^.

The Control Group (CG) continued with conventional monitoring, carried out in the Primary Health Units, through clinical care, according to the Primary Health Care protocol of Belo Horizonte Council^12^. The users also received an educational booklet containing information on the pathophysiology of diabetes, prevention of acute and chronic complications, importance of diet and the practice of physical activity and foot care. Finally, these users received two phone calls, performed by a nurse, for the application of the measuring instruments of the study (Figure 2).

### Measuring instruments

In order to collect the socio-demographic and economic profile data of the participants, a questionnaire was developed and applied which included the following: age, marital status, gender, income, duration of disease, education and work situation. For the evaluation of the nutritional status, the weight was obtained using a digital scale (Marte^(r))^ and the height measured using a portable stadiometer (Altura Exata^(r))^ to the nearest 0.1cm. Regarding the evaluation of metabolic control, the glycohemoglobin examination was previously scheduled and collected in the Primary Health Unit. A glycohemoglobin level lower or equal to 7.0% (53mmol/mol) was defined as an indicator of good metabolic control^13^.

Finally, to check the frequency of the self-care actions recommended for disease control, for the previous seven days, the Diabetes Self-care Questionnaire - ESM was applied, which consists of eight items. The responses are presented in a multiple-choice scale and the total score ranges from zero to eight points, with scores higher than or equal to five indicating an appropriate frequency of following an eating plan and practicing physical activity^14^.

### Sample size

To calculate the sample, the usual values ​​were established: i) 95% confidence interval (α=0.05), ii) power of 80%, iii) a minimum standardized difference of 1.5 standard deviations to be detected between the means of the self-care scores of the two groups at the three moments, iv) an intraclass correlation coefficient of 0.05 and v) mean size of 100 users (target population) in each of the eight health centers. From these values, an expression was used to consider the design effect (cluster randomized) to calculate the number of users in each health center (cluster)^15^. After considering a participant attrition rate of 20%, 219 users were included in the trial.

### Randomization

The Primary Health Units were allocated to the Control Group (CG) and Experimental Group (EG), from a draw using a 1:1 ratio, with randomization being performed to ensure that the two groups could be considered homogeneous regarding the measure of self-care. The health units were numbered 1 to 8 and, through the drawing of these numbers (via computer program and without replacement), each unit was allocated to one of the groups, alternately, giving a total of four in each group. The draw process and allocation of the Primary Health Units to the study groups was carried out by a Statistics professional who was not involved in the recruitment phase of the health centers. At the end, each group consisted of four PHUs each, with the control group containing 110 users and the experimental group 109 users in total.

### Blinding

A nurse, blinded to the group allocation of the users, during the period prior to the start of the study, applied the measuring instrument, remaining blinded until all the instruments had been completed. The data analysis, in turn, was made by a Statistics professional also blinded to the group to which the users were allocated.

### Data collection

Data collection was performed by a nurse and was divided into three phases: 1) prior to the start of the telephone intervention or conventional care (T0), 2) after 3 months (T3) and 3) after six months (T6). For both groups, experimental and control, at the T0 moment the first contact with the users, through face to face interviews, was performed in the Basic Health Units, at which time the sociodemographic, economic, anthropometric and glycohemoglobin data were collected. The Diabetes Self-care Questionnaire was applied at the three moments (T0, T3 and T6), with the application being made via phone call at the last two moments.

### Statistical analysis

The data were processed, using double entry for the control of possible errors, in the SPSS, version 20, program. The statistical analyzes of the data were performed using the Minitab, version 15, software. The following statistical tests were used: 1) Student's t-test, for the comparison between groups, 2) Kolmogorov-Smirnov test to verify the validity of the normality assumption, and 3) Wilcoxon and Mann-Whitney tests, when the assumption of normality was not valid.

The evaluation of the effect of time on each group, in turn, was performed by using a repeated measures analysis of variance for balanced data, with Bonferroni correction used in the tests of multiple comparisons. In the case of comparisons between proportions, the chi-square test or Fisher's exact test was used. For all analyzes a 5% significance level was considered.

### Ethical issues

The development of the study adhered to the national and international ethical standards for research involving human subjects, according to Resolution 196/96 of the National Health Council. The project that gave rise to this study was approved by the Research Ethics Committee of the Municipal Health Department (Authorization No. 0731.0.203.410-12A). All participants, after clarification regarding the aims and criteria of participation, signed two copies of the terms of consent form.

## Results

**Figure 3:**
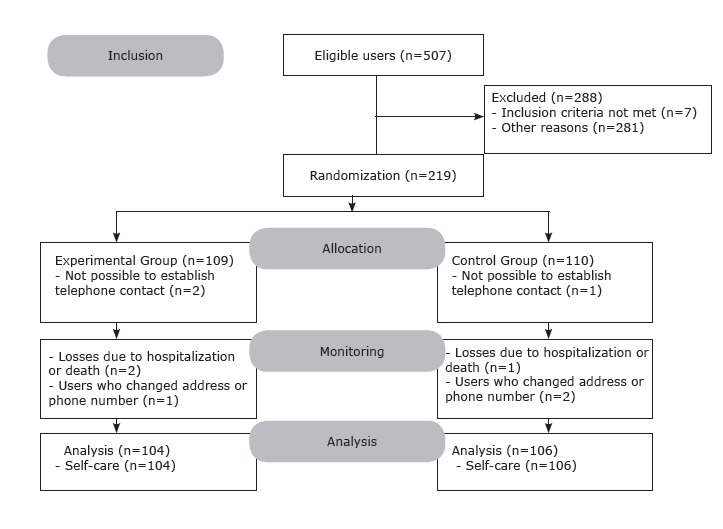
Consort Diagram. Belo Horizonte, MG, Brazil, 2012

 Of the total of 219 users of the sample, nine did not participate in at least two telephone interventions, with it not being possible to establish telephone contact with two users of the EG and one user of the CG (incorrect phone number or the user did not answer the calls), two users hospitalized (CG=1 and EG=1) and one user having died (EG=1). In the course of the research, three users (CG=2 and EG=1) changed their address and/or telephone number. Figure 3 shows that, at the end of six months, 210 users completed the study and were analyzed, these being 104 of the EG and 106 of the CG.

Table 1 shows that both groups, experimental and control, were mainly constituted by women and had a mean age over 60 years. Users that did not work and users who had a partner made up the majority in both the experimental and control groups. The majority of users of both groups had complete or incomplete elementary education and average monthly income of less than two minimum wages. With respect to the clinical and anthropometric data, both groups presented mean glycohemoglobin levels above 7.0% and mean BMI above 25kg/m^2^ (Table 1). As shown in Table 1, the control and experimental groups were homogeneous, according to the sociodemographic and economic variables, except for education. The control group had a lower percentage of illiterate subjects and a higher percentage of users with complete or incomplete higher education, compared to the experimental group.

**Table 1:** Distribution of the sample at the initial moment (T0) of the study, regarding the sociodemographic and economic variables of users with diabetes. Belo Horizonte, MG, Brazil, 2012

Variable		Control group (n=106)	Experimental group (n=104)	P value
Gender %*				0.213
	Male	38 (35.9)	28 (26.9)	
	Female	68 (64.1)	76 (73.1)	
Education %*				0.010^†^
	Illiterate	6 (5.7)	19 (18.3%)	
	Elementary (complete or not)	75 (70.8)	71 (68.2%)	
	High School (complete or not)	20 (18.8)	13 (12.5%)	
	Higher (complete or not)	5 (4.7)	1 (1.0%)	
Work situation %*				1.000
	Active	21 (19.8)	21 (20.2%)	
	Inactive	85 (80.2)	83 (79.8%)	
Marital status %*				0.592
	With partner	65 (61.3)	59 (56.7%)	
	Without partner	41 (38.7)	45 (43.3%)	
Mean age, in years (SD)‡		62.9±10.3	62.9±10.2	1.000
Mean income in MW§ (SD)‡		1.8±1.8	1.8±1.4	1.000
Glycohemoglobin in % (SD)‡		8.12±2.18	8.02±2.07	0.734
BMI, in kg/m2 (SD)‡		28.43±5.01	29.11±5.59	0.355

* Percent; †Fisher's exact test; ‡standard deviation; §minimum wage=R$ 622.00 (US$=304.90)

Regarding the ESM scores, a progressive increase of the values can be observed in the EG at T0, T3 and T6. Conversely, for the CG, there was an improvement in the mean score from T0 to T3, however, from T3 to T6, this group presented a decrease in the mean score (Table 2). 

**Table 2:** Means of ESM scores in the evaluations at T0, T3 and T6 and comparison of the effect on the mean score of self-care (T6-T0) in the Experimental and Control Groups. Belo Horizonte, MG, Brazil, 2012

Self-care	Experimental group Mean±SD*	Control group Mean±SD†	95% CI† for the effect T6-T0
T0	3.52±0.71	3.55±0.76	
T3	5.20±0.94	4.34±1.10	
T6	5.53±0.80	4.10±1.18	
Effect T6-T0	1.97±0.96	0.57±1.53	1.03; 1.78

*Standard deviation; †Confidence Interval

As shown in Table 2, the mean effect on self-care in the EG can be considered statistically different from the mean effect in the CG (p=0.000), considering the period between T0 and T6. With a 95% confidence interval, the mean effect on the score of the EG was 1.03 to 1.78 higher than for the CG.

With regard to the characteristics of the phone calls to the experimental group, the mean length of contact during the calls was 14.0 minutes (95% CI 13.6 to 14.4). The percentages of users who were contacted on the first, second and third attempts were 43.5, 27.8 and 12.0%, respectively, while for 16.7% of the users, it took four or more contact attempts (95% CI 10.4 to 25.3).

## Discussion

The data from this study show that the telephone intervention was effective, since the mean effect of the self-care scores, measured before and after the program for the users who received the telephone intervention, presented substantial change compared with the control group. Thus, these findings corroborate the results of an experimental study involving 62 users with diabetes, in which the group that underwent telephone intervention for 12 weeks presented better self-care scores compared to the control group^16^. Complementarily, review studies point out the effectiveness of this educational strategy, particularly with regard to the improvement of self-care for the reduction of glycohemoglobin and blood pressure values compared or associated with conventional care^7^ ^,^ ^17^ ^-^ ^18^.

It was observed that the systematic monitoring and the use of an approach centered on the users and their needs, in which they are the transforming agents of their reality, contributed to improve the practices of physical activity and following the food plan, among the users of the experimental group. These data can be related to the fact that the phone calls make dialogue and reflection possible, therefore, encouraging the co-responsibility of the users with diabetes in relation to their own health^6^. Furthermore, the low educational level, evidenced among the users with diabetes studied, raised the need to use appropriate and easy to understand language, respecting their capabilities and limitations. Studies reinforce the role of the health professional as a facilitator of learning, awakening the users to their potential to make informed, conscious decisions regarding self-care, thus achieving the autonomy and skill necessary for their own health care management^2^ ^,^ ^6^.

It should be noted, however, that the users of both groups at T0 in this study had a mean self-care score below 5 points, indicating that the frequency of physical activity and following the food plan were inadequate, according to the recommendations for the control of diabetes. Similarly, an intervention study performed in Brazil with users with diabetes, which used the same measuring instrument, also evidenced low self-care scores at the start of the study^19^.

For the users of the control group, despite the slight improvement in self-care scores obtained over the six-month period, the results suggest that conventional care provided by the primary care units was insufficient to achieve or sustain the score of 5 points. These findings lead to reflection on the fragility of the educational practices performed by primary health care teams, suggesting that these actions are ineffective or have limitations in promoting diabetes self-care^20^. Therefore, this reinforces the need to seek innovative strategies, such as the telephone intervention, aiming to act in a complementary way to boost the self-care promotion actions of these services^4^.

The use of the telephone is considered an effective method to approach the users in their homes or communities, with flexibility in the hours and optimization of the time. Consequently, this means that currently it provides an educational strategy capable of reaching large numbers of users who have difficulties, such as geographical and financial barriers, in accessing the health services^20^ ^-^ ^21^. It should be noted that the telephone access of the population has significant increased, driven by the growth in mobile telephones, considering that 75.2% of Brazilians over 10 years of age had a mobile phone in 2013, an increase of 131.4% in this contingent compared to 2005^22^. Thus, it is believed that the telephone intervention demonstrates the potential reach and viability to address the user with diabetes, promoting continuity of care and the educational activities provided^5^ ^-^ ^6^.

During the planning and execution of this study, other factors that could influence the effectiveness of telephone intervention were also considered, such as the training of the two nurses involved in the study (the first being responsible for performing the telephone intervention and the second for the application of measuring instruments). Accordingly, it was sought to standardize the language and the quality of the information provided, designing a script to guide the calls and organize the days and times of the calls to ensure the success of the contact.

Some limitations must be pointed out. These include the short monitoring period for the evaluation of the educational program that ideally should be more than 12 months, to better assess the effect of the intervention, and the absence of analysis of the clinical variables, such as the glycohemoglobin level. Thus, these limitations can be addressed in future studies.

## Conclusion

The telephone intervention performed with users with type 2 diabetes mellitus, tested as an educational strategy, was effective in promoting self-care related to physical activity and following a diet plan. It is hoped that this study will lead to future developments, implementing the telephone intervention for diabetes education in Primary Health Care, and that this strategy can be evaluated for its contribution in the clinical management of type 2 diabetes mellitus.

## References

[1] Yonah B, Esterson MC, John DP, Nihal T, Meredith H (2014). A Systematic Review of Innovative Diabetes Care Models in Low-and
Middle-Income Countries (LMICs). J Health Care Poor Underserved.

[2] Pereira DA, Costa NMSC, Sousa ALM, Jardim PCBV, Zanini CRO (2012). The effect of educational intervention on the disease knowledge of
diabetes mellitus patients Rev. Latino-Am. Enfermagem.

[3] Imazu MFM, Faria BN, Arruda GO, Sales CA, Marcon SS (2015). Effectiveness of individual and group interventions for people with
type 2 diabetes Rev. Latino-Am. Enfermagem.

[4] Koutsouris D, Lazakidou A, Vellidou L, Iliopoulou D (2014). The use of telephone monitoring for diabetic patients theory and
practical implications. Smart Homecare Technol TeleHealth.

[5] Aliha JM, Asgari M, Khayeri F, Ramazani M, Farajzadegan Z, Javaheri J (2013). Group Education and Nurse-Telephone Follow-Up Effects on Blood Glucose
Control and Adherence to Treatment in Type 2 Diabetes Patients. Int J Prev Med.

[6] Torres HC, Reis IA, Roque C, Faria P (2013). Monitoramento telefônico como estratégia educativa para o autocuidado
das pessoas com diabetes na atenção primária. Cienc Enferm.

[7] Hunt CW. (2015). Technology and diabetes self-management: An integrative
review. World J Diabetes.

[8] Goodarzi M, Ebrahimzadeh I, Rabi A, Saedipoor B, Jafarabadi MA (2012). Impact of distance education via mobile phone text messaging on
knowledge, attitude, practice and self efficacy of patients with type 2 diabetes
mellitus in Iran. J Diabetes Metab Disord.

[9] Freire P (2002). Pedagogia da autonomia: saberes necessários à prática educativa.

[10] Instituto Brasileiro de Geografia e Epidemiologia (2014). Pesquisa Nacional de Saúde 2014: Percepção do estado de saúde, estilo de vida
e doenças crônicas.

[11] Sociedade Brasileira de Diabetes (2013). Diretrizes da Sociedade Brasileira de Diabetes 2012-2013.

[12] Ministério da Saúde (2013). Caderno de Atenção Básica. Estratégia para o cuidado da pessoa com doença
crônica: diabetes mellitus.

[13] Executive Summary: Standards of Medical Care in Diabetes-2011 (2011). Diabetes. Care.

[14] Torres HC, Franco LJ, Stradioto MA, Hortale AH, Schall VT (2009). Avaliação estratégica de educação em grupo e individual no programa
educativo em diabetes. Rev Saúde Pública.

[15] Campbell MK, Thomson S, Ramsay CR, Maclennan GS, Grimshaw JM (2004). Sample size calculator for cluster randomised trials. Computers Biol Med.

[16] Jaleh MA, Mina A, Feridone K, Majid R, Ziba F, Javad J (2013). Group Education and Nurse-Telephone Follow-Up Effects on Blood Glucose
Control and Adherence to Treatment in Type 2 Diabetes Patients. Int J Prev Med.

[17] Calvin KL, Da Tao (2014). Does the use of consumer health information technology improve
outcomes in the patient self-management of diabetes A meta-analysis and narrative
review of randomized controlled trials. Int J Med Inform.

[18] Vasconcelos HCA, Freitas RWJF, Marinho NBP, Damasceno MMC, Araújo AL, Lima FET (2013). Eficácia de intervenções que utilizam o telefone como estratégia para
o controle glicêmico revisão integrativa da literatura Texto
Contexto. Enferm.

[19] Nundya S, Dickb JJ, Solomonc MC, Peeka ME (2013). Developing a behavioral model for mobile phone-based diabetes
interventions. Patient Educ Couns.

[20] Carneiro ACLL, Souza V, Godinho LK, Faria ICM, Silva KL, Gazzinelli MF (2012). Educação para a promoção da saúde no contexto da atenção
primária. Rev Panam Salud Publica.

[21] Liu L, Ogwu S (2012). A Meta-Analysis of Mobile Health and Risk Reduction in Patients with
Diabetes Mellitus Challenge and Opportunity. J Mobil Technol Med.

[22] Instituto Brasileiro de Geografia e Estatística (IBGE) (2013). Pesquisa Nacional por Amostra de Domicílios. Acesso à internet e à televisão
e posse de telefone móvel celular para uso pessoal.

